# Characterization and Antimicrobial Activity of Biodegradable Active Packaging Enriched with Clove and Thyme Essential Oil for Food Packaging Application

**DOI:** 10.3390/foods9081117

**Published:** 2020-08-13

**Authors:** Shubham Sharma, Sandra Barkauskaite, Brendan Duffy, Amit K. Jaiswal, Swarna Jaiswal

**Affiliations:** 1 School of Food Science and Environmental Health, College of Sciences and Health, Technological University Dublin—City Campus, Grangegorman, D07H6K8 Dublin, Ireland; Shubham.Sharma@TUDublin.ie (S.S.); C16713221@mytudublin.ie (S.B.); swarna.jaiswal@TUDublin.ie (S.J.); 2 Environmental Sustainability and Health Institute (ESHI), Technological University Dublin—City Campus, Grangegorman, D07H6K8 Dublin, Ireland; 3 Centre for Research in Engineering and Surface Technology (CREST), FOCAS Institute, Technological University Dublin—City Campus, Kevin Street, D08NF82 Dublin, Ireland; brendan.duffy@TUDublin.ie

**Keywords:** active food packaging, antimicrobial activity, antibiofilm activity, clove essential oils, thyme essential oil, poly (lactide), poly (butylene adipate-co-terephthalate)

## Abstract

Bioactive packaging contains natural antimicrobial agents, which inhibit the growth of microorganisms and increase the food shelf life. Solvent casting method was used to prepare the Poly (lactide)-Poly (butylene adipate-co-terephthalate) (PLA-PBAT) film incorporated with the thyme oil and clove oil in various concentrations (1 wt%, 5 wt% and 10 wt%). The clove oil composite films depicted less green and more yellow as compared to thyme oil composite films. Clove oil composite film has shown an 80% increase in the UV blocking efficiency. The tensile strength (TS) of thyme oil and clove oil composite film decreases from 1.35 MPs (control film) to 0.96 MPa and 0.79, respectively. A complete killing of *S. aureus* that is a reduction from 6.5 log CFU/mL to 0 log CFU/mL was observed on the 10 wt% clove oil incorporated composite film. Clove oil and thyme oil composite film had inhibited *E. coli* biofilm by 93.43% and 82.30%, respectively. Clove oil composite film had exhibited UV blocking properties, strong antimicrobial activity and has high potential to be used as an active food packaging.

## 1. Introduction

Active packaging is a novel method used to prolong the shelf-life of perishable foods, maintain or improve the quality and safety of prepared foods due to its interaction with the product. Besides, active packaging for consumable goods has the potential to reduce the addition of active compounds into foods, reduction in the movement of particles from film to food and localisation activities could introduce the pathogenic microorganism into the product [1]. Also, the world focus has now shifted to biodegradable and non-biodegradable polymers such as poly-hydroxy alkanoates (PHA), polyethylene (PE), poly (lactide) (PLA), bio-based polyethylene terephthalate (PET), poly (butylene adipate-co-terephthalate) (PBAT) based packaging. Poly(lactide) (PLA) and Poly (butylene adipate-co-terephthalate) (PBAT) are the biodegradable polymers, and various studies were conducted on the blend of PLA and PBAT [2]. Biodegradable polymer incorporated with an active compound could ensure the food safety, quality and monitor environmental health [3].

Depending on the types of additives incorporated into the film, active packaging can be categorised into chemoactive and bioactive. Chemoactive packaging has an impact on the chemical composition of the food product and gaseous atmosphere inside a pack. It includes ethylene scavengers, modified atmosphere packaging, and moisture control systems. On the other hand, bioactive packaging contains antioxidant and antimicrobial agents within the package that interact with biological molecules and may inhibit the growth of various microorganisms [3] A study by Azadbakht et al. [4] used *Eucalyptus globulus* essential oil in chitosan to examine the antimicrobial activity in the packaging of sliced sausages. The results showed that the log reduction value could be improved by increasing essential oil concentration. The application of essential oils such as cinnamon oil, clove oil, thyme oil in active packaging can be used in the forms of films and coatings. Films are usually thin sheets that are made beforehand and can be used as covers, wrappers, layer separation or packaging for various foods. On the other hand, coatings are defined as films that can be applied onto the surface of an edible product [5].

In recent years, there is a growing concern towards chemical active packaging due to the use of synthetic additives and materials that can cause adverse health effects or make packaging unsustainable for recycling leading to high waste volume. Due to growing consumer demand for natural products, synthetic additives become replaced by substance of natural origin, such as essential oils and plant extracts [6]. Natural antioxidants can interact with the food product and package headspace resulting in the protection of the food products from oxidation.

Antioxidant active packaging can either release antioxidants into the food and the package or absorb oxygen and other compounds from the food or its surroundings. Also, active packaging containing natural essential oil, antioxidants like clove oil, green tea oil, thyme oil is a cost-saving alternative that also has the potential to eliminate food safety risks [7]. Therefore, the natural plant extracts and essential oils play a significant role in the antimicrobial/antioxidant activity of the active packaging. The effectiveness of active packaging containing thyme essential oil/β-cyclodextrin ε-polylysine nanoparticles (TCPNs) was tested [8]. The results showed that TCPNs incorporated into gelatin nanofibers significantly improved the antimicrobial properties against bacteria such as *Campylobacter jejuni*.

There are several examples of essential oils and their constituents incorporated into active films. Chitosan films containing *Eucalyptus globulus* essential oil were developed for the packaging of sliced sausages that have a high potential to reduce the antimicrobial activity and control the food-borne contamination in food systems [4]. Another study carried out by Perdones et al. [9] showed chitosan-based coatings containing lemon essential oil were very effective in delaying the ripening process in strawberries due to their reduced respiration rate. It was also determined that after seven days of storage, the aroma of lemon essential oil did not have any impact on the organoleptic properties of strawberries. The antibacterial activity of these newly developed packaging system was attributed to the chemical composition of used essential oil [4,9].

Clove (*Syzygium aromaticum*) is commonly used in the seasoning of food. Recent research showed that clove essential oil can kill numerous bacteria and some fungi, and the antimicrobial activity is attributable to eugenol, oleic acids and lipids found in its essential oils. Jirovetz et al. [10] found that clove essential oil comprises 23 constituents, among them eugenol (76.8%), followed by β-caryophyllene (17.4%), α-humulene (2.1%), and eugenyl acetate (1.2%) were the main components. Pinto et al. [11] had also evaluated clove oil by GC-MS and the found a total of 19 components. The oil contains 85.3% of the phenylpropanoid compound (eugenol) [11]. Moreover, Shrivastava et al. [12] had analysed clove oil from India and Madagascar. They found 35 constituent in the Madagascar clove oil and 28 constituent in the Indian clove oil where the major content was eugenol (82.6%), β-caryophyllene (7.2%), eugenyl acetate (6%), α-bergamontene (0.2%), α-humulene (0.8%), ϒ-cadinene (0.2%), iso-eugenol-I (0.1%), selinene (0.3%) and allo-aromadendrene (0.1%) [12]. Razafimamonjison et al. [13] had evaluated the clove essential oil from bud, lead and stem. In all essential oils, they also found the major constituent as eugenol in bud (72.08–82.36%), stem (87.52–96.65%) and leaf (75.04–83.58%). Eugenyl acetate was also found in bud (8.61–21.32%), leaf (0–1.45%) and stem (0.07–2.53%). Moreover, β-caryophyllene are found more in leaf (11.65–19.53%), than in bud essential oils (2.76–8.64%) and in stem essential oils (1.66–9.72%) [13].

Thyme (*Thymus vulgaris*) essential oil is used as flavor enhancer for foods and beverages and known to possess antimicrobial and antioxidant properties, which are due to presence of various constituents such as thymol, carvacrol and cymene [14]. In a study carried out by Porte et al. [15] reported that thyme oil has thirty-nine constituents, where major constituents of the oil were thymol (44.7%), p-cymene (18.6%) and γ-terpinene (16.5%). Various studies have analysed thyme essential oil and found the main components as thymol (55.3%), p-cymene (11.2%), carvacrol (8.7%, and β-caryophyllene (4.2%). The other contents found were isoborneol (2.3%), γ-terpinene (3.4%), acetovanillone (1.7%) and linalool (1.7%) [16,17]. Tohidi et al. had analysed thyme oil by gas chromatography–mass spectrometry (GC–MS) and found 38 compounds having major elements as thymol (12.4–79.74%), carvacrol (4.37–42.14%), p-cymene (0.8–12.86%) and geraniol (0.3–22.44%) [18].

In the present study, essential oils (thyme oil and clove oil) have been incorporated as an antimicrobial agent in a PLA/PBAT (poly (lactide)/poly (butylene adipate-co-terephthalate) blend film for food packaging application. Effect of thyme oil and clove oil on the structural, functional, mechanical, antimicrobial and biofilm inhibition properties of the composite films was studied. To the best of author’s knowledge, this is the first report to incorporate thyme oil and clove oils into the biodegradable polymer (PLA/PBAT) blend film and investigate their potential as an active food packaging.

## 2. Materials and Methods

### 2.1. Materials

PBAT (poly (butylene adipate-co-terephthalate) (m.p. 110–120 °C, the density of 1.26 g/cm^3,^ Ecoworld PBAT003;) and Poly (lactide) (PLA, the weight-average molecular weight of 200 kDa, Synterra BF 2004) were purchased from Helian Polymer (Belfeld, Netherland). Ethanol was purchased from Merck KGaA (Germany) and Chloroform was obtained from Sigma Aldrich (Ireland). Clove essential oil (Mol wt. 150.2 g/mol, relative density 1.04 g/cm^3^ at 25 °C) and Thyme essential oil (Mol wt. 164.2 g/mol, relative density 0.917 g/cm^3^ at 25 °C) of natural origin (100% purity) were purchased from a local supermarket, Dublin, Ireland. Tryptic Soy Broth (TSB) and Tryptic Soy Agar [8] powder were purchased from Sigma Aldrich (Ireland). Foodborne pathogenic bacteria, *Staphyloccocus aureus* (ATCC 25923) and *Escherichia coli* (NCTC 9001) were used in this study. All of these strains were grown in TSB media and stored at 4 °C before further testing.

### 2.2. Preparation of Composite Films

By using a solvent casting method, the essential oils (thyme and clove) were incorporated into biodegradable films of PLA-PBAT [19]. The blending ratio of PLA:PBAT was taken as 98:2 [20]. In 100 mL chloroform various concentrations of essential oil (1 wt%, 5 wt%, and 10 wt% of polymer resin) were pipetted out. To make up the equal volume in all the beaker ethanol was added in the samples accordingly. 4 g of polymer resin (3.92 g of PLA and 0.08 g of PBAT) and 50 wt% of glycerol [21] were added and mixed using a magnetic stirrer at 23 ± 2 °C for 24 h. A control sample of the PLA/PBAT film was prepared using the same method, followed by the addition of only ethanol (without any essential oil).

After the stirring of 24 h the solubilised mixture was poured onto a Teflon coated glass plate (24 cm × 30 cm) and spread evenly with a bend glass rod and allowed to dry at the room temperature for 24 h. The prepared films were peeled off from the glass plates and placed into the incubator at 25 °C and 50% relative humidity (RH) for 48 h. The Control films prepared was named as Poly (lactide)-Poly (butylene adipate-co-terephthalate) (PLA-PBAT) [22]. The thyme containing films were PLA/PBAT-Thyme ^1%^, PLA/PBAT-Thyme ^5%^, and PLA/PBAT-Thyme ^10%^. Clove containing films were named as PLA/PBAT-Clove ^1%^, PLA/PBAT-Clove ^5%^, and PLA/PBAT-Clove ^10%^.

### 2.3. Characterisation of Films

#### 2.3.1. Surface Colour and Optical Properties

ColorQuest XE (Hunter Lab) spectrophotometer was used to determine the surface colour of composite films. The colour values were taken in terms of L (+lightness, −darkness), a (+redness, −greenness), and b (+yellowness, -blueness). White colour plate (L = 93.97, a = −0.88, b = 1.21) was used as a standard background. For each film the readings are taken in triplicate from different locations. The mean value with the standard deviation of the readings was considered. The total colour difference which determines the difference between the colour values of standard tile and film samples (∆E) was considered using an equation:

ΔE = [(ΔL)^2^ + (Δa)^2^ + (Δb)^2^] ^1/2^

where ΔL, Δa, and Δb are the differences between standard colour plate, each colour value and the colour value of the film samples, respectively [22,23].

The optical properties such as the light transmittance spectrum was observed using UV-Vis spectrophotometer. The rectangular films of (3 cm × 7 cm) were cut and mounted between two spectrophotometer magnetic cells. The light transmittance spectrum of the composite film was measured between the wavelength of 200–700 nm. UV-light barrier property was obtained by the percent transmittance at 280 nm (T_280_) and transparency of the films were determined at 600 nm (T_600_).

Fourier-transform infrared spectrometer (FTIR) (Thermo Scientific) was used to determine the infrared spectrum of absorption of the composite films. FTIR was operated at the resolution of 4 cm^−1^. The film sample of 4 cm × 4 cm were cut and positioned on the ray exposing stage (crystal plate). The spectrum was recorded at wavenumber of 4000–400 cm^−1^. The readings were taken in triplicates from the surface. The functional groups present in the composite films were determined by the peak at specific wavenumber.

#### 2.3.2. Thickness and Tensile Properties

The thickness of the composite films was determined by Digital micrometre (Mitutoyo, Japan) having 0.001 mm resolution. Five readings were taken from the random positions. The mean values and standard deviation were considered of these readings.

Mechanical properties such as tensile strength (TS) and elongation at break (EB) of the composite films were measured by using standard ASTM D 882–88 method [24] by Instron Universal Testing (Model 5565, Instron Engineering Corporation, Canton, MA, USA). The composite films were cut into rectangular strips of 3 cm × 15 cm. Grip length of 50 mm and a crosshead speed of 50 mm/min using a 500 N load cell set in Instron Instrument were used to operate at room temperature until the sample broke at a certain point. The Tensile Strength was calculated using the Equation:

TS = F/A

where *F* is the maximum force (N) required to separate the sample, and A is the initial cross-sectional area (m^2^) of the composite films [22].

Percentage elongation at break (EB) is the ratio between changed film length and initial length of the samples [25]. The EB was calculated using the following formula:

EB (%) = [(X_f_ − X_o_)/X_o_] × 100

where X_o_ is the initial grips separation (50 mm) of samples, X_f_ is the film elongation at the moment of failure.

#### 2.3.3. Surface Hydrophobicity

Water contact angle (WCA) determines the interaction of the film surface with the liquid interphase by using a dynamic contact angle analyser (FTA-200 system) [26]. It evaluates the surface is hydrophobic or hydrophilic in nature. Rectangular films of 3 cm × 8 cm placed on the stainless-steel platform having the water contact angle analyser attached. With the help of a micro-syringe a drop of distilled water approximately 10 μL was dropped on the film surface. The interaction of the drop on the surface of the film was observed by taking a picture with a high-speed camera and analysed it by the image processed by computer. Three readings were taken from the different location. Also, the experiment was performed in triplicates.

#### 2.3.4. Antibacterial Activity

The Japanese Industrial Standard (JIS Z 2801:2000) [27] was used to determine the antibacterial efficacy of the composite films incorporated with thyme oil and clove oils against *Staphylococcus aureus* (ATCC 25923) and *Escherichia coli* (NCTC 9001). The kinetic study was performed using square pieces of the composite films (5 cm × 5 cm). Each film was sterilised each side using UV light for 20 min. Films were then placed on the aluminium plate (5.5 cm × 5.5 cm) and aseptically transferred into a sterilised petri dish containing filter paper which was wetted with autoclaved water. The initial bacterial inoculum 10^6^ CFU/mL was prepared aseptically for the test. 200 µL of the test inoculum was then pipetted onto three pieces of each film, leaving one film as a positive control. Each petri dish containing the sample was covered with a lid and then incubated with the test inoculum at the temperature of 37 °C and relative humidity (RH) of >90%. Films were tested at 0 h, 4 h, 8 h, 12 h and 24 h. One set of inoculated samples was immediately tested after 0 h by placing the samples in a sterilised stomacher bag. 20 mL of Maximum Recovery Diluent (MDR) was poured and mixed gently by the stomacher (AGB Scientific-Lab blender 400) for 40–45 s. For the viable cell counts the samples were taken from the MRD culture, appropriately diluted and plated on Tryptic Soy Agar plates. The above process is repeated to determine the viable cell count after 4 h, 8 h, 12 h and 24 h samples and the control sample after their incubation.

#### 2.3.5. Biofilm Inhibition

To study the biofilm inhibition of the essential oil composite film common food borne pathogen *E. coli* (NCTC 9001) was used. Three square pieces of 4 cm × 4 cm of each film (one set for positive control and two sets for biofilm inhibition) were cut and sterilised using UV light for 20 min. Films were then placed on the aluminium plate (5 cm × 5 cm) and transferred into a sterilised petri dish containing filter paper which was wetted with autoclaved water. 400 µL of the prepared test inoculum (10^6^ CFU/mL) was put on the film and incubated at 37 °C and relative humidity (RH) of >90% for 72 h. After the incubation, each film was rinsed with sterile water three times. The films were then stained with 1% (*w*/*v*) crystal violet (500 µL) for 45 min [28]. The test film samples were again washed thrice with the sterile distilled water. Biofilms were quantified by eluting crystal violet with 95% ethanol (500 µL) for 10 min and transferring 100 µL aliquots to 96 well plates. The absorbance was then determined at 600 nm to determine the percent inhibition of *E. coli* biofilm.

### 2.4. Statistical Analysis

All measurements are determined in triplicates. Statistical differences between multiple sample comparisons were evaluated by analysis of variance (ANOVA) and multiple comparisons (Fischer’s least significant difference test) using STATGRAPHICS Centurion XV software (Stat Point Technologies Inc. Warrenton, VA, USA). Differences were considered to be significant if the value of *p* < 0.05. All results are stated as mean ± standard deviation.

## 3. Results and Discussion

### 3.1. Surface Colour

To make the food product look more attractive, is played by the appearance of the active food packaging. The surface colour and transparency are the major factor to adopt the food packaging. The film appeared to be homogeneous, smooth and flexible. L (+lightness, −darkness), a (+redness, −greenness), and b (+yellowness, −blueness) values of the composite films incorporated with the essential value steadily increased (Table 1 and Table 2). With the increase in the concentration of both the essential oils (thyme and clove) the lightness was observed to decrease significantly (*p* < 0.05). Also, the *a*-value of the thyme oil composite film significantly decreased by 1.2 folds while, for the clove oil composite film decreased by 2.07 folds as the oil concentration increases from 1 wt% to 10 wt%.

In addition, the yellowness (*b*-value) of films increased significantly in the clove oil composite film. It was observed that the yellowness of the thyme composite film increased by 1.4 folds with the increase in the concentration of the oil, whereas, in the clove oil composite oil increased by 3.7 folds. The greenness and yellowness values of the clove oil composite film (PLA/PBAT-clove oil films) are significantly different from each other and depicted the highest a and b values, which means less green and more yellow as compared to PLA/PBAT-thyme oil films. The phenolic compounds present in the clove oil could be a possible reason for the increase in the yellowness in the PLA/PBAT-clove oil films [29]. The thyme oil composite film had shown decrement in the total colour difference (∆E values) while, of PLA/PBAT-clove blend film ∆E values increased as the oil concentration increased (Table 1 and Table 2). Similar results have been observed by various studies [30], after the incorporation of the clove oil in polymer film has observed the transformation of colour from bluish to yellowish. Furthermore, Ejaz [31] et al., had observed the decrease in transparency with the increase in the concentration of clove oil in the film matrix due to the presence of the colouring elements in essential oil and heterogenous network of the film.

### 3.2. Optical Properties

**Figure 1:**
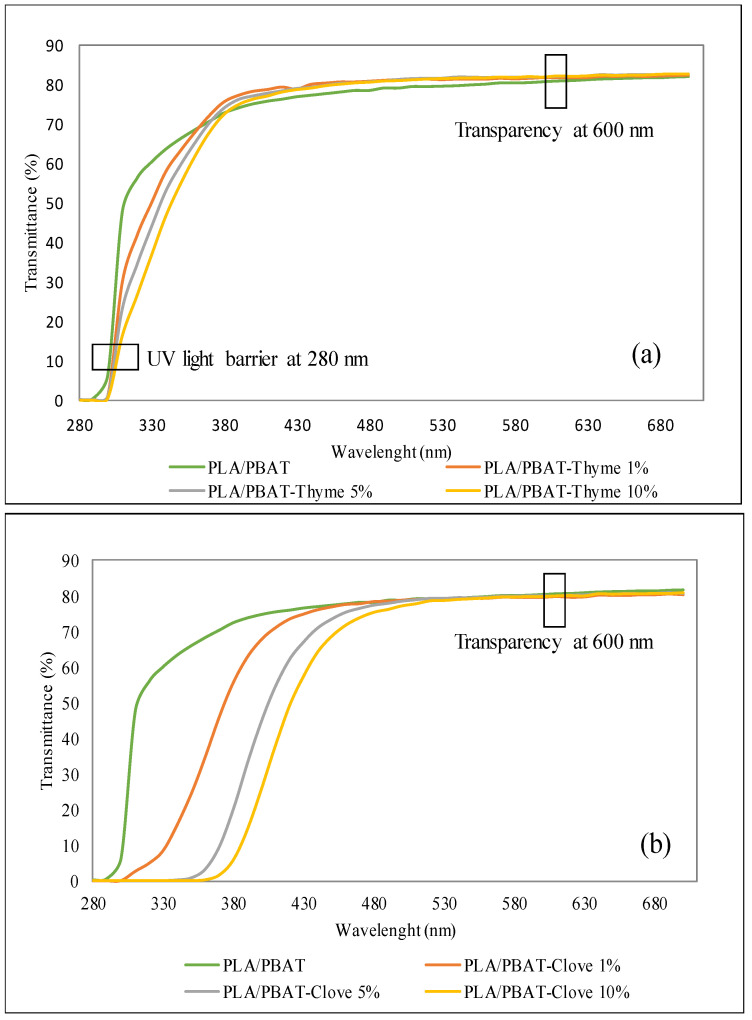
Light transmittance spectra of PLA/PBAT incorporated with (**a**) thyme oil and (**b**) clove oil composite film.

Transparency of the active food packaging plays a very important role in the acceptability of film by the consumers. Another optical property which plays a significant role in the food industry is the UV barrier property of the film. The film must be able to prevent the oxidation of the food induced by the UV light and increase the shelf life of the food. The transmission spectrum of light was measured at 280–700 nm to determine the optical properties of the composite film (Figure 1a,b). The control film (PLA/PBAT) showed the higher transmittance at 300 nm, whereas the essential oil incorporated film showed no or low transmittance at 300 nm.

As seen in the Figure 1b, the transmittance of the PLA/PBAT-thyme oil films had shown a remarkable increase above 280 nm whereas, the PLA/PBAT-clove oil films increased above 380 nm. Furthermore, the incorporation of the essential oil (clove and thyme oil) decreased the transmittance at 280 nm (T_280_), which indicates the superior UV barrier property of the film significantly (*p* < 0.05). The decrease in the transmittance at 280 nm was due to the interruption in the passage of light through the film surface [32,33]. The T_280_ (for UV light) depicted that for the PLA/PBAT-Thyme^10%^ UV light blocking property increased by 20% with respect to the control film while, of the PLA/PBAT-Clove^10%^ increased by 80% (Table 1 and Table 2). A very high concentration of the phenolic compound in the clove oil absorbed UV light which results for the best UV blocking properties for the clove oil composite film. These results align with various studies in which a UV barrier property had been observed by the incorporation of clove essential oil in the film matrix. Mulla [33] et al., observed a significant increase in the UV barrier property after the incorporation of clove oil. Similarly, Ejaz [31] et al., had observed the improvement in the UV blocking property of the composite film.

T_600_ (for visible light) depicts the transparency of the composite films. No significant difference was observed between control film and 1 wt% thyme composite film, and between 5 wt% and 10 wt% thyme composite film. The transmittance of light at 600 nm of the control film is observed to be 72.46%. On the incorporation of thyme oil (10 wt%) transmittance value is observed as 81.28% (Table 1) whereas by the incorporation of clove oil (10 wt%) it becomes 65.53% (Table 2). The value observed suggests that on the incorporation of thyme oil the transparency of the film increased, while clove oil incorporation had decreased the transparency of the film. Teixeria [34] et al., had also observed the decrease in the transparency due to the incorporation of clove oil. Sanuja [35] et al., had also observed the increase in the opaqueness and decrease in the transparency of the packaging film due to the incorporation of the clove oil. The transparency of the PLA/PBAT-clove oil films is observed to decrease due to the presence of phenolic compounds which led to the yellowish tint composite film. Therefore, due to the high property of UV blocking PLA/PBAT-clove films could be used as an active packaging material.

### 3.3. FTIR Analysis

**Figure 2:**
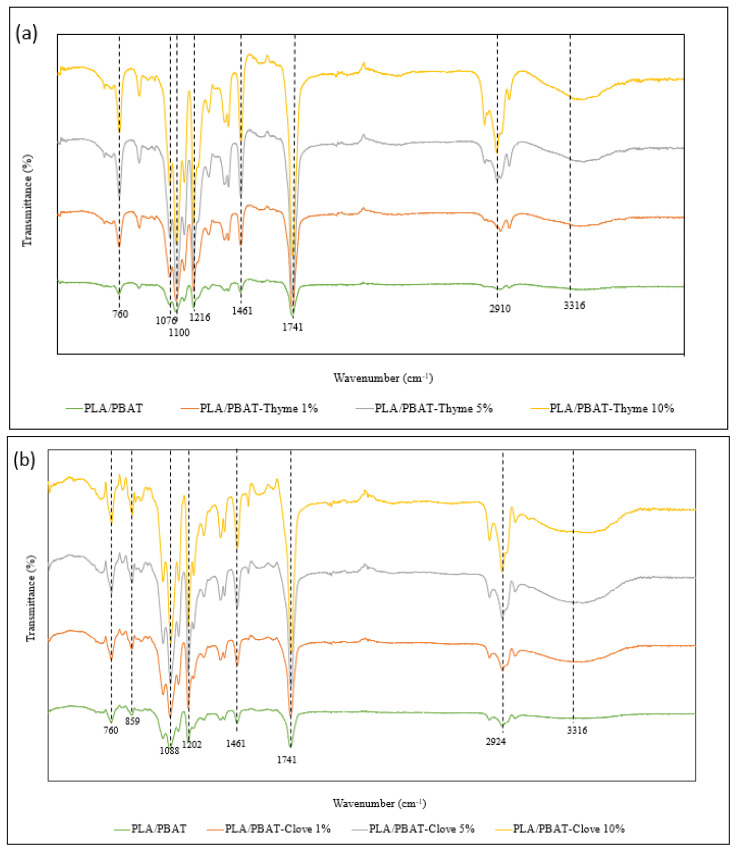
FTIR spectra of PLA/PBAT incorporated with (**a**) thyme oil and (**b**) clove oil composite film.

For the qualitative analysis of the chemical properties of the composite film Fourier transform infrared spectroscopy (FTIR) was performed. The FTIR spectra has shown the peaks demonstrating the chemical composition of the composite film (Figure 2). The overlapping peaks at 727,884 and 1445 cm^−1^, demonstrated the presence of the aromatic compounds with C-H and C-C stretch (in the ring). The peaks determined in the range of 1073–1079 cm^−1^ stands for the aliphatic amines with C-N stretch. Range of 1176–1180 cm^−1^ stands for the C-O vibrational stretching, which depicts the presence of alcohols, carboxylic acids, esters and ethers. The existence of alkanes in the composite films was determined by their C-H vibrational stretching in the range of 1353–1364 cm^−1^. The occurrence of O-H stretching resulted in a wide peak at 3265–3316 cm^−1^ wavelength range indicating the presence of alcohols and phenols. It was observed that as the concentration of the essential oil in the composite film increased the functional groups like O-H had shown shorter peaks, whereas, the peaks exhibiting C-N, C-O, -CHO had become sharper (Figure 2). Correa-Pacheco [36] et al., had mentioned that essential oils are unstable compounds and are susceptible to transformation, polymerization and oxidation, which could be a possibility of the chemical changes occurring in the PLA/PBAT composite films.

### 3.4. Thickness of the Films

Digital micrometer was used to measure the thickness of the composite films. It was observed that the thickness of the composite films increases with the increase in the concentration of the essential oil (thyme oil and clove oil). The thickness of the thyme oil increased by 3.08 folds (113.33 µm of PLA/PBAT-Thyme^10%^) with respect to the control PLA/PBAT film (36.71 µm) while, the thickness of the clove oil composite film increased by 2.90 folds (106.67 µm of PLA/PBAT-Clove^10%^) depicting significant difference in the values (Table 3 and Table 4). Similar results are seen in studies where the thickness increased with the incorporation of essential oil in the active packaging film [37,38]. This could be due to the distribution pattern of the essential oil in the matrix of the film.

### 3.5. Tensile Properties of the Films

Universal Testing Machine (Zwick/Roell-A730271) was used to determine the mechanical properties (tensile strength (TS) and elongation at break (EB)) of the essential oil incorporated composite film. The tensile strength of the composite films was observed to decrease with the incorporation of the essential oils (thyme oil and clove oil). The tensile strength of the control PLA/PBAT film was observed as 1.35 MPa (Table 3 and Table 4). No significant difference in the control film, 1 wt% and 5 wt% thyme oil composite film was observed. As the thyme oil in the composite film increases from 1 wt% to 10 wt% the TS decreases from 1.52 MPa to 0.96 MPa, respectively, whereas, the increase in the clove oil concentration in the composite film the TS decreases from 0.94 MPa to 0.79 MPa. This shows a significant difference in the 10 wt% thyme oil composite film and 10 wt% clove oil composite films from other films. The increase in the concentration of the oil in the composite oil could weaken the structure as the stronger polymer-polymer interaction is replaced by a weaker polymer-oil interaction [39,40].

The flexibility of the film is depicted by the elongation at break (EB). The EB is observed to increase with the increase in the concentration (Table 3 and Table 4). A significant difference between the 10 wt% thyme oil, 10 wt% clove oil composite film and the control film was observed. The EB of the control film PLA/PBAT was observed to be 5.63%. With the increase in the thyme oil concentration in the composite film (PLA/PBAT-Thyme^10%^) the EB increases to 16.51%, while, for the increase in the clove oil concentration in the composite film (PLA/PBAT-Clove^10%^) the EB increases to 25.67%. The improved flexibility of the composite films is due to a better interaction between PLA and PBAT polymers and a presence of the essential oil.

Many studies show that the incorporation of the essential oil influences the TS depending on the interaction of the oil with the composite film matrix [39]. Bonilla [41] et al., had incorporated basil and thyme essential oil in chitosan film and studied that the film had become more fragile with increased strechability [41]. Furthermore, Chen [42] et al., had studied 14.13% decrease in TS and 26.64% increase in EB after the incorporation of clove oil in poly(vinyl alcohol) active film. Hosseini [43] et al., had also stated that the destabilization process could take place during the drying of the film and could lead to the reduction in TS.

### 3.6. Water Contact Angle (WCA)

Water Contact angle (WCA) is used to determine the surface hydrophobicity. WCA indicates the interaction of the film with the liquids which is important for the application of the film in the food industry [44]. The surface is determined to be hydrophobic if the contact angle higher than 65° [22,45]. The control PLA/PBAT film has shown WCA as 61.61° (Table 3 and Table 4). No significant difference was observed in the WCA value of the control, 1 wt% and 5 wt% of the essential oil (thyme oil and clove oil) incorporated films, whereas the WCA value of higher concentration (10 wt% of thyme and clove oil) composite film showed significant difference. As the thyme oil concentration in the composite film increased from 1 wt% to 10 wt% the WCA increased from 72.57 to 80.57°, whereas as the clove oil concentration increased from 1 wt% to 10 wt% in the composite film the WCA increased from 62.24 to 74.74°. Therefore, the incorporation of the clove oil (10 wt%) and thyme oil (1 wt%, 5 wt% and 10 wt%) had increased the hydrophobicity of the film, which could play a significant role in the increase in the quality of the active packaging.

### 3.7. Antibacterial Activity

**Figure 3:**
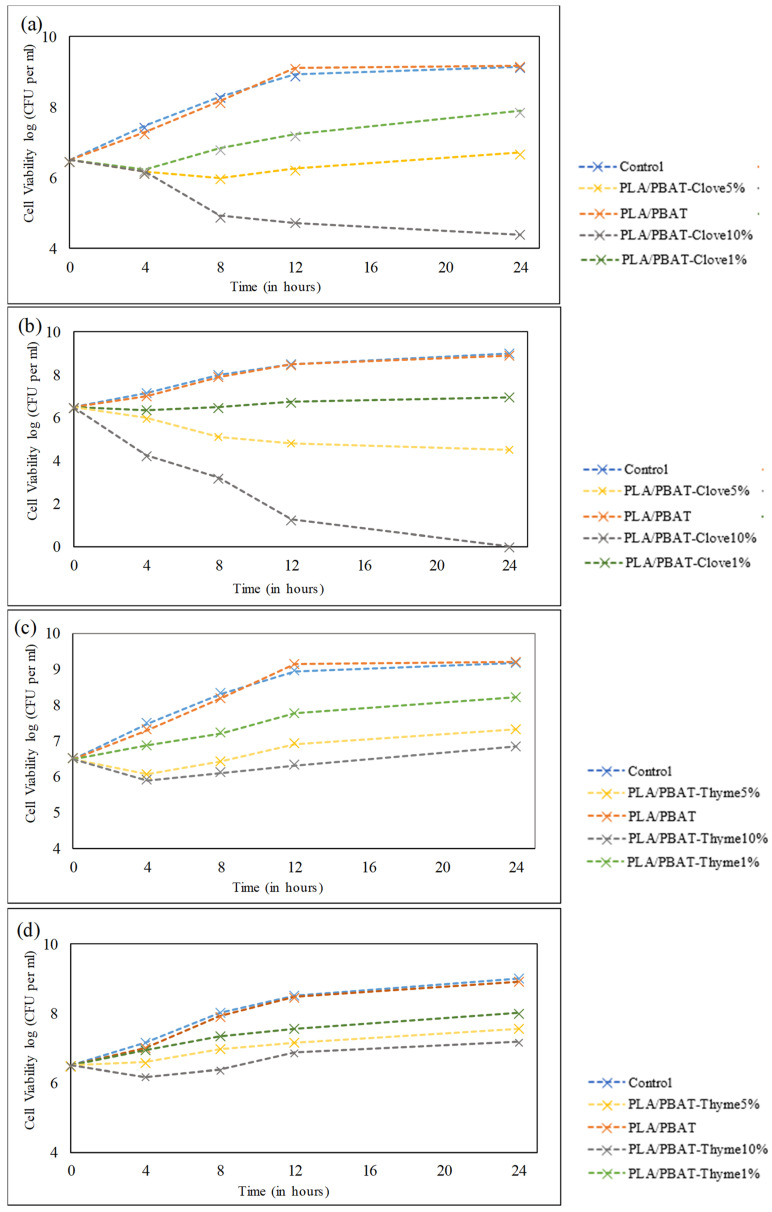
Antimicrobial efficiency of composite film. (**a**) effect of clove oil composite film on *E. coli*, (**b**) effect of clove oil composite film on *S. aureus* (**c**) effect of thyme oil composite film on *E. coli* and (**d**) effect of thyme oil composite film on *S. aureus*.

Antibacterial active packaging prevents the growth of bacteria on the surface of the food due to direct contact of the packaging material. The antibacterial efficiency of the essential oil (thyme and clove oil) incorporated composite film was determined against *E. coli* (Gram negative bacteria) and *S. aureus* (Gram positive bacteria). The incorporation of the clove oil in the composite film had demonstrated significant antibacterial activity. It was also observed in Figure 3c,d that, as the thyme oil composite film doesn’t demonstrate antibacterial activity for 24 h. Though 5 wt% thyme oil composite film or the thymol content shows antimicrobial activity for the first 4 h against *E. coli* whereas, 10 wt% thyme oil composite film shows antibacterial activity for 8 h against *S. aureus* and 12 h against *E. coli*. Thyme oil composite film (10 wt%) slightly reduces the growth of *E. coli* from 6.5 log CFU/mL to 6.32 log CFU/mL in 12 h after that it reaches to 6.84 log CFU/mL.

As shown in Figure 3a, the 1 wt% clove oil composite film has shown antibacterial effect against *E. coli* and *S. aureus* in the first 4 h, whereas 5 wt% clove oil composite have shown antibacterial activity against *E. coli* up till 12 h, after that it has not shown antibacterial activity. However, in the presence of 10 wt% clove oil composite film (PLA/PBAT-Clove^10%^) *E. coli* growth had reduced from 6.5 log CFU/mL to 4.4 log CFU/mL. However, an exceptional antibacterial activity has been observed by 10 wt% clove oil composite film against *S. aureus*. In presence of 5 wt% clove oil composite film the *S. aureus* growth had reduced from 6.5 log CFU/mL to 4.5 log CFU/mL. 10 wt% clove oil composite film had shown a complete killing of *S. aureus* by reducing growth from 6.5 log CFU/mL to 0 log CFU/mL. It has been reported that the incorporation of essential oil including clove oil has higher bacterial inhibition efficacy against Gram positive bacteria [46]. Mulla [33] et al., had also studied strong antibacterial efficacy of clove essential oil against *S. typhimurium* and *L. monocytogenes*. Moreover, Mupalla et al., (2014) had observed that the clove oil film had shown promising antibacterial activity. Also, the films were observed to be more active against *S. aureus* than *B. cereus* [47].

### 3.8. Biofilm Inhibition

**Figure 4:**
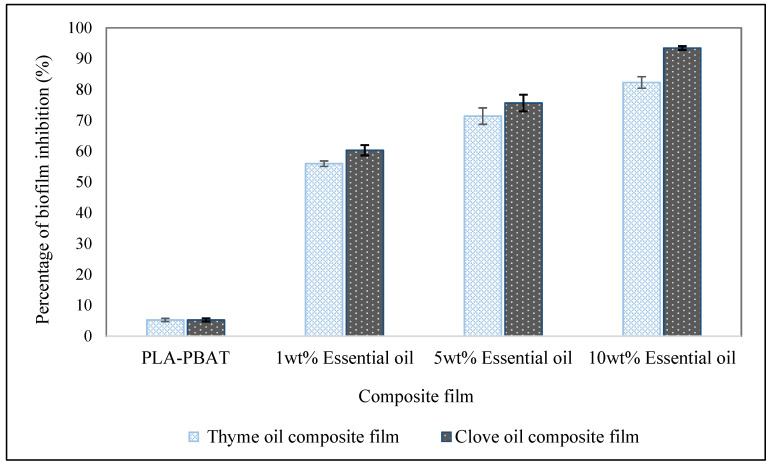
Biofilm inhibition of PLA/PBAT-Thyme oil composite film and PLA/PBAT-Clove oil composite film against *E. coli*.

Aggregation of bacteria on the surface led to the formation of the biofilm. Biofilms on the food contact surfaces causes critical problems in the food industry. A biofilm study was observed against *E. coli* for 72 h. As shown in Figure 4, the control film shows minimal biofilm inhibition (5.27%). The thyme oil composite film (1 wt%) had inhibited biofilm by 55.96%, whereas 1 wt% clove oil composite film had inhibited *E. coli* growth by 60.31%. As the concentration rises to 5 wt%, thyme oil composite film had inhibited biofilm growth by 71.39% and clove oil composite film had inhibited biofilm growth of *E. coli* by 75.65%.

However, a significant inhibition of *E. coli* biofilm growth was observed by clove oil composite film (10 wt%). 10 wt% clove oil composite film had inhibited the biofilm by 93.43%. Moreover, clove oil was more effective than thyme oils as they contain higher concentrations of eugenol, 180 mg [48]. Therefore, the results signify that the high concentration (10 wt%) of essential oils (thyme oil or clove oil) inhibit the formation of the biofilm (*E. coli*) on all tested composite films, though the percentage inhibition of clove oil (93.43% inhibition) was higher than thyme oil (82.30% inhibition). Cui [49] et al., had also studied that the incorporation of clove oil-loaded chitosan nanoparticle had inhibited *E. coli* biofilm up to 99.99%.

## 4. Conclusions

Thyme oil and clove oils were incorporated into the biodegradable polymer (PLA/PBAT) blend film and their potential as an active food packaging was investigated. Incorporation of clove oil and thyme oil had a very high influence on the composite film properties such as optical, morphological, mechanical properties, antibacterial efficacy and biofilm inhibition property. The clove oil composite films depicted pale yellow film with less transparency and high UV-light barrier property as compared to thyme oil composite films. Clove oil composite film has shown 80% UV barrier property. Significant decrease in the tensile strength by the incorporation of the essential oil has been observed. The tensile strength decreases from 1.35 (control film) to 0.96 MPa (PLA/PBAT-Thyme^10%^) and 0.79 MPa (PLA/PBAT-Clove^10%^). However, as the concentration of essential oil increases, the water contact angle (WCA) of the composite films has increased significantly. Also, a very high antibacterial property had been shown by clove oil composite film. Clove oil composite film had reduced *E. coli* growth by 2.1 log CFU/mL. Also, a complete killing of *S. aureus* that is, a reduction from 6.5 log CFU/mL to 0 log CFU/mL was observed by 10 wt% clove oil composite film. The thyme oil composite film (10 wt%) had inhibited biofilm by 82.30% against *E. coli* whereas, clove oil composite film (10 wt%) had inhibited the *E. coli* biofilm by 93.43%. Thus, the clove oil composite film has demonstrated strong UV barrier, antibacterial and biofilm inhibition property, which can avoid the undesirable photochemical reaction, adherence and growth of pathogenic bacteria which can increase the shelf life of the packed food. Therefore, clove oil composite film can be used as an active packaging.

## Figures and Tables

**Table 1:** Surface colour, UV barrier and transparency of composite films with Thyme oil.

Film	Lightness (L)	Redness, Greenness (a)	Yellowness, Blueness (b)	Total Colour Difference (∆E)	Transmittance (T_280_ (%))	Transmittance (T_600_ (%))
PLA/PBAT	91.56 ± 0.36 ^a^	−1.08 ± 0.02 ^c^	1.34 ± 0.03 ^a^	2.42 ± 0.35 ^d^	0.05 ± 0.00 ^b^	72.46 ± 0.63 ^a^
PLA/PBAT-Thyme^1%^	93.21 ± 0.08 ^b^	−1.17 ± 0.02 ^b^	1.31 ± 0.04 ^a^	0.97 ±0.24 ^a^	0.04 ± 0.01 ^a^	73.43 ± 1.45 ^a^
PLA/PBAT-Thyme^5%^	92.93 ± 0.05 ^c^	−1.27 ± 0.01 ^a^	1.69 ± 0.03 ^b^	1.22 ± 0.04 ^b^	0.04 ± 0.01 ^a^	81.28 ± 2.63 ^b^
PLA/PBAT-Thyme^10%^	92.95 ± 0.03 ^d^	−1.30 ± 0.02 ^a^	1.89 ± 0.07 ^c^	1.29 ± 0.05 ^c^	0.04 ± 0.01 ^a^	81.28 ± 3.32 ^b^

The letters (a–d) at each concentration specifies groups that are significantly different (*p* < 0.05).

**Table 2:** Surface colour, UV barrier and transparency of composite films with Clove oil.

Film	Lightness (L)	Redness, Greenness (a)	Yellowness, Blueness (b)	Total Colour Difference ∆E	Transmittance (T_280_ (%))	Transmittance (T_600_ (%))
PLA/PBAT	91.56 ± 0.54 ^a^	−1.08 ± 0.02 ^d^	1.34 ± 0.03 ^a^	2.42 ± 0.03 ^b^	0.05 ± 0.00 ^c^	72.46 ± 1.07^c^
PLA/PBAT-Clove^1%^	93.12 ± 0.96 ^a^	−1.41 ± 0.01 ^c^	2.08 ± 0.05 ^b^	1.33 ± 0.04 ^a^	0.02 ± 0.00 ^b^	71.63 ± 0.35^c^
PLA/PBAT-Clove^5%^	92.55 ± 0.79 ^a^	−2.06 ± 0.03 ^b^	4.23 ± 0.25 ^c^	3.55 ± 0.04 ^c^	0.01 ± 0.00 ^a^	69.63 ± 0.68 ^b^
PLA/PBAT-Clove^10%^	92.14 ± 1.18 ^a^	−2.99 ± 0.02 ^a^	7.78 ± 1.35 ^d^	7.15 ± 0.04 ^d^	0.01 ± 0.00 ^a^	65.53 ± 0.93^a^

The letters (a–d) at each concentration specifies groups that are significantly different (*p* < 0.05).

**Table 3:** Thickness, tensile properties and hydrophobicity of the thyme oil composite films.

Film	Thickness (µm)	Tensile Strength (TS (MPa))	Elongation at Break (EB (%))	Water Contact Angle (WCA (Degree))
PLA/PBAT	36.71 ± 4.16 ^a^	1.35 ± 0.03 ^b^	5.63 ± 0.41 ^b^	61.61 ± 2.82 ^a^
PLA/PBAT-Thyme^1%^	46.67 ± 5.73 ^a,b^	1.52 ± 0.08 ^b^	3.10 ± 0.38 ^a^	72.57 ± 1.54 ^b^
PLA/PBAT–Thyme^5%^	63.33 ± 10.17 ^b^	1.26 ± 1.26 ^b^	5.84 ± 0.25 ^b^	74.06 ± 3.00 ^b^
PLA/PBAT–Thyme ^10%^	113.33 ± 14.59 ^c^	0.96 ± 0.08 ^a^	16.51 ± 0.47 ^c^	80.57 ± 2.28 ^c^

The letters (a–d) at each concentration specifies groups that are significantly different (*p* < 0.05).

**Table 4:** Thickness, tensile properties and hydrophobicity of the clove oil composite films.

Film	Thickness (µm)	Tensile Strength (TS (MPa))	Elongation at Break (EB (%))	Water Contact Angle (WCA (Degree))
PLA/PBAT	36.71 ± 4.58 ^a^	1.35 ± 0.03 ^c^	5.63 ± 0.41 ^a^	61.61 ± 2.82 ^a^
PLA/PBAT-Clove^1%^	46.67 ± 3.59 ^a^	0.94 ± 0.08 ^b^	39.97 ± 0.09 ^d^	62.24 ± 5.75 ^a^
PLA/PBAT-Clove^5%^	73.33 ± 7.07 ^b^	0.89 ± 0.06 ^a, b^	27.58 ± 0.36 ^c^	64.69 ± 2.15 ^a^
PLA/PBAT-Clove ^10%^	106.67 ± 16.68 ^c^	0.79 ± 0.03 ^a^	25.67 ± 0.52 ^b^	74.74 ± 4.11 ^b^

The letters (a–d) at each concentration specifies groups that are significantly different (*p* < 0.05).

## References

[1] Schaefer D., Cheung W.M. (2018). Smart packaging: Opportunities and challenges. Procedia CIRP.

[2] Weng Y.X., Jin Y.J., Meng Q.Y., Wang L., Zhang M., Wang Y.Z. (2013). Biodegradation behavior of poly (butylene adipate-co-terephthalate) (PBAT), poly (lactic acid) (PLA), and their blend under soil conditions. Polym. Test..

[3] Brockgreitens J., Abbas A. (2016). Responsive food packaging: Recent progress and technological prospects. Compr. Rev. Food Sci..

[4] Azadbakht E., Maghsoudlou Y., Khomiri M., Kashiri M. (2018). Development and structural characterization of chitosan films containing Eucalyptus globulus essential oil: Potential as an antimicrobial carrier for packaging of sliced sausage. Food Packag. Shelf Life.

[5] Ribeiro-Santos R., Andrade M., de Melo N.R., Sanches-Silva A. (2017). Use of essential oils in active food packaging: Recent advances and future trends. Trends Food Sci. Technol..

[6] Vinceković M., Viskić M., Jurić S., Giacometti J., Kovačević D.B., Putnik P., Jambrak A.R. (2017). Innovative technologies for encapsulation of Mediterranean plants extracts. Trends Food Sci. Technol..

[7] Domínguez R., Barba F.J., Gómez B., Putnik P., Kovačević D.B., Pateiro M., Lorenzo J.M. (2018). Active packaging films with natural antioxidants to be used in meat industry: A review. Food Res. Int..

[8] Lin L., Zhu Y., Cui H. (2018). Electrospun thyme essential oil/gelatin nanofibers for active packaging against *Campylobacter jejuni* in chicken. LWT.

[9] Perdones Á., Escriche I., Chiralt A., Vargas M. (2016). Effect of chitosan–lemon essential oil coatings on volatile profile of strawberries during storage. Food Chem..

[10] Jirovetz L., Buchbauer G., Stoilova I., Stoyanova A., Krastanov A., Schmidt E. (2006). Chemical composition and antioxidant properties of clove leaf essential oil. J. Agric. Food Chem.

[11] Pinto E., Vale-Silva L., Cavaleiro C., Salgueiro L. (2009). Antifungal activity of the clove essential oil from *Syzygium aromaticum* on *Candida, Aspergillus* and *dermatophyte* species. J. Med. Microbiol..

[12] Srivastava A.K., Srivastava S.K., Syamsundar K.V. (2005). Bud and leaf essential oil composition of *Syzygium aromaticum* from India and Madagascar. Flav. Frag. J..

[13] Razafimamonjison G., Jahiel M., Duclos T., Ramanoelina P., Fawbush F., Danthu P. (2014). Bud, leaf and stem essential oil composition of *Syzygium aromaticum* from Madagascar, Indonesia and Zanzibar. Int. J. Basic Appl. Sci..

[14] Nzeako B., Al-Kharousi Z.S., Al-Mahrooqui Z. (2006). Antimicrobial activities of clove and thyme extracts. Sultan Qaboos Univ. Med. J..

[15] Porte A., Godoy R.L. (2008). Chemical composition of *Thymus vulgaris* L. (Thyme) essential oil from the Rio de Janeiro state, Brazil. J. Serbian Chem. Soc..

[16] Gedikoğlu A., Sökmen M., Çivit A. (2019). Evaluation of *Thymus vulgaris* and *Thymbra spicata* essential oils and plant extracts for chemical composition, antioxidant, and antimicrobial properties. Food Sci. Nutr..

[17] Imelouane B., Amhamdi H., Wathelet J.P., Ankit M., Khedid K., El Bachiri A. (2009). Chemical composition and antimicrobial activity of essential oil of thyme (*Thymus vulgaris*) from Eastern Morocco. Int. J. Agric. Biol..

[18] Tohidi B., Rahimmalek M., Arzani A. (2017). Essential oil composition, total phenolic, flavonoid contents, and antioxidant activity of Thymus species collected from different regions of Iran. Food Chem..

[19] Shankar S., Rhim J.W. (2016). Tocopherol-mediated synthesis of silver nanoparticles and preparation of antimicrobial PBAT/silver nanoparticles composite films. LWT-Food Sci. Technol..

[20] Wang L.F., Rhim J.W., Hong S.I. (2016). Preparation of poly (lactide)/poly (butylene adipate-co-terephthalate) blend films using a solvent casting method and their food packaging application. LWT-Food Sci. Technol..

[21] Zhen Z., Ying S., Hongye F., Jie R., Tianbin R. (2011). Preparation, characterization and properties of binary and ternary blends with thermoplastic starch, poly (lactic acid) and poly (butylene succinate). Polym. Renew. Resour..

[22] Sharma S., Jaiswal A.K., Duffy B., Jaiswal S. (2020). Ferulic acid incorporated active films based on poly (lactide)/poly (butylene adipate-co-terephthalate) blend for food packaging. Food Packag. Shelf Life.

[23] Shankar S., Rhim J.W. (2018). Preparation of antibacterial poly (lactide)/poly (butylene adipate-co-terephthalate) composite films incorporated with grapefruit seed extract. Int. J. Biol. Macromol..

[24] ASTM D882-18 (2018). Standard Test Method for Tensile Properties of Thin Plastic Sheeting.

[25] Petroudy S.R.D. (2017). Physical and mechanical properties of natural fibers. Advanced High Strength Natural Fibre Composites in Construction.

[26] Al-Naamani L., Dobretsov S., Dutta J. (2016). Chitosan-zinc oxide nanoparticle composite coating for active food packaging applications. Innov. Food Sci. Emerg. Technol..

[27] JIS, Z Japanese Standards Association (2008). Antimicrobial Products–Test for Antimicrobial Activity and Afficacy.

[28] Jaiswal S., Bhattacharya K., McHale P., Duffy B. (2015). Dual effects of β-cyclodextrin-stabilised silver nanoparticles: Enhanced biofilm inhibition and reduced cytotoxicity. J. Mater. Sci. Mater. Med..

[29] Pattanasiri T., Taparhudee W., Suppakul P. (2017). Anaesthetic efficacy of clove oil-coated LDPE bag on improving water quality and survival in the Siamese fighting fish, β-splendens, during transportation. Aquac. Int..

[30] Arfat Y.A., Ahmed J., Ejaz M., Mullah M. (2018). Polylactide/graphene oxide nanosheets/clove essential oil composite films for potential food packaging applications. Int. J. Biol. Macromol..

[31] Ejaz M., Arfat Y.A., Mulla M., Ahmed J. (2018). Zinc oxide nanorods/clove essential oil incorporated Type B gelatin composite films and its applicability for shrimp packaging. Food Packag. Shelf Life.

[32] Arfat Y.A., Benjakul S., Prodpran T., Sumpavapol P., Songtipya P. (2014). Properties and antimicrobial activity of fish protein isolate/fish skin gelatin film containing basil leaf essential oil and zinc oxide nanoparticles. Food Hydrocoll..

[33] Mulla M., Ahmed J., Al-Attar H., Castro-Aguirre E., Arfat Y.A., Auras R. (2017). Antimicrobial efficacy of clove essential oil infused into chemically modified LLDPE film for chicken meat packaging. Food Control.

[34] Teixeira B., Marques A., Pires C., Ramos C., Batista I., Saraiva J.A., Nunes M.L. (2014). Characterization of fish protein films incorporated with essential oils of clove, garlic and origanum: Physical, antioxidant and antibacterial properties. LWT-Food Sci. Technol..

[35] Sanuja S., Agalya A., Umapathy M. (2014). Studies on magnesium oxide reinforced chitosan bio-nanocomposite incorporated with clove oil for active food packaging application. Int. J. Polym. Mater..

[36] Correa-Pacheco Z.N., Black-Solís J.D., Ortega-Gudiño P., Sabino-Gutiérrez M.A., Benítez-Jiménez J.J., Barajas-Cervantes A., Hurtado-Colmenares L.B. (2020). Preparation and characterization of bio-based PLA/PBAT and cinnamon essential oil polymer fibers and life-cycle assessment from hydrolytic degradation. Polymers.

[37] De Medeiros J.A.S., Blick A.P., Galindo M.V., Alvim I.D., Yamashita F., Ueno C.T., Shirai M.A., Grosso C.R.F., Corradini E., Sakanaka L.S. (2019). Incorporation of oregano essential oil microcapsules in Starch-Poly (Butylene Adipate Co-Terephthalate) (PBAT) Films. Macromol. Symp..

[38] Salarbashi D., Tajik S., Ghasemlou M., Shojaee-Aliabadi S., Noghabi M.S., Khaksar R. (2013). Characterization of soluble soybean polysaccharide film incorporated essential oil intended for food packaging. Carbohydr. Polym..

[39] Atarés L., Chiralt A. (2016). Essential oils as additives in biodegradable films and coatings for active food packaging. Trends Food Sci. Technol..

[40] Shojaee-Aliabadi S., Hosseini H., Mohammadifar M.A., Mohammadi A., Ghasemlou M., Ojagh S.M., Khaksar R. (2013). Characterization of antioxidant-antimicrobial κ-carrageenan films containing Satureja hortensis essential oil. Int. J. Biol. Macromol..

[41] Bonilla J., Atarés L., Vargas M., Chiralt A. (2012). Effect of essential oils and homogenization conditions on properties of chitosan-based films. Food Hydrocoll..

[42] Chen C., Xu Z., Ma Y., Liu J., Zhang Q., Tang Z., Xie J. (2018). Properties, vapour-phase antimicrobial and antioxidant activities of active poly (vinyl alcohol) packaging films incorporated with clove oil. Food Control.

[43] Hosseini S.F., Rezaei M., Zandi M., Farahmandghavi F. (2015). Bio-based composite edible films containing Origanum vulgare L. essential oil. Ind. Crops Prod..

[44] Yao Z.C., Chen S.C., Ahmad Z., Huang J., Chang M.W., Li J.S. (2017). Essential oil bioactive fibrous membranes prepared via coaxial electrospinning. J. Food Sci..

[45] Vogler E.A. (1998). Structure and reactivity of water at biomaterial surfaces. Adv. Colloid Interface Sci..

[46] Song N.B., Lee J.H., Al Mijan M., Song K.B. (2014). Development of a chicken feather protein film containing clove oil and its application in smoked salmon packaging. LWT-Food Sci. Technol..

[47] Muppalla S.R., Kanatt S.R., Chawla S., Sharma A. (2014). Carboxymethyl cellulose–polyvinyl alcohol films with clove oil for active packaging of ground chicken meat. Food Packag. Shelf Life.

[48] Khalil A.A., ur Rahman U., Khan M.R., Sahar A., Mehmood T., Khan M. (2017). Essential oil eugenol: Sources, extraction techniques and nutraceutical perspectives. RSC Adv..

[49] Cui H., Bai M., Rashed M.M., Lin L. (2018). The antibacterial activity of clove oil/chitosan nanoparticles embedded gelatin nanofibers against *Escherichia coli* O157: H7 biofilms on cucumber. Int. J. Food Microbiol..

